# A Framework for White Blood Cell Segmentation in Microscopic Blood Images Using Digital Image Processing

**DOI:** 10.1007/s12575-009-9011-2

**Published:** 2009-06-11

**Authors:** Farnoosh Sadeghian, Zainina Seman, Abdul Rahman Ramli, Badrul Hisham Abdul Kahar, M-Iqbal Saripan

**Affiliations:** 1 Department of Computer and Communication Systems Engineering, Faculty of Engineering, Universiti Putra Malaysia, 43400 Serdang, Selangor, Malaysia; 2 Department of Pathology, Faculty of Medicine and Health Sciences, Universiti Putra Malaysia, 43400 Serdang, Selangor, Malaysia; 3 Sapura Secured Technologies, Section 10, Wangsa Maju, Kuala Lumpur, Malaysia

**Keywords:** White blood cell segmentation, Active contours, Snake algorithm, Zack thresholding

## Abstract

Evaluation of blood smear is a commonly clinical test these days. Most of the time, the hematologists are interested on white blood cells (WBCs) only. Digital image processing techniques can help them in their analysis and diagnosis. For example, disease like acute leukemia is detected based on the amount and condition of the WBC. The main objective of this paper is to segment the WBC to its two dominant elements: nucleus and cytoplasm. The segmentation is conducted using a proposed segmentation framework that consists of an integration of several digital image processing algorithms. Twenty microscopic blood images were tested, and the proposed framework managed to obtain 92% accuracy for nucleus segmentation and 78% for cytoplasm segmentation. The results indicate that the proposed framework is able to extract the nucleus and cytoplasm region in a WBC image sample.

## 1. Introduction

White blood cells (WBC) or leukocytes play a significant role in the diagnosis of different diseases, and therefore, extracting information about that is valuable for hematologists. In the past, digital image processing techniques have helped to analyze the cells that lead to more accurate, standard, and remote disease diagnosis systems. However, there are a few complications in extracting the data from WBC due to wide variation of cells in shape, size, edge, and position. Moreover, since illumination is imbalanced, the image contrast between cell boundaries and the background varies depending on the condition during the capturing process.

This study is focusing on WBC segmentation using L2 microscopic images. Our goal is to segment the WBC nucleuses and cytoplasm using a framework that has been developed using digital image processing. The use of image processing techniques have developed rapidly in the last few years, to the point where hematologists can use blood images to automatically process blood slides for the first screening in detecting diseases. These techniques can help to find cell counts in human blood automatically and also can provide information about ratio of nucleus versus cytoplasm to identify and classify different types of WBCs such as neutrophil, basophil, lymphocyte, etc. Therefore, in this paper, we present a proposed framework that consists of several methods that integrates together for nucleus segmentation and cytoplasm extraction.

Many works have been conducted in the area of general segmentation methods. Among the common segmentation methods are edge and border detection, region growing, filtering, mathematical morphology, and watershed clustering. Ritter et al. [1] presented a fully automatic method for segmentation and border identification of all objects that do not overlap the boundary in an image taken from a peripheral blood smear slide. In their work, pale tips of protuberances are lost. Ongun et al. [2] did segmentation by morphological preprocessing followed by the snake-balloon algorithm. Jiang et al. [3] proposed a WBC segmentation scheme on color space images using feature space clustering techniques, scale-space filtering for nucleus extraction, and watershed clustering for cytoplasm extraction. Leyza et al. [4] used morphological operators and examined the scale-space properties of toggle operator to improve segmentation accuracy. Scotti [5] presented the automatic morphological method that is based on the morphological analysis of WBCs. Their proposed system extracts the morphological indexes (lymphocytes). Kumar et al. [6] used teager energy operator for segmentation, nucleus based on the edges, which are detected effectively by teager energy operator but it required at least a weak edge to exist between red blood cell (RBC) and the background. For cytoplasm segmenting, they used a simple morphological method. Cseke introduced multi-step segmentation scheme [7], which implements the automatic thresholding method proposed by Otsu [8].

The remainder of this paper is organized as follows. In **Section 2**, segmentation algorithms and the framework are explained. In **Section 3**, results obtained by the proposed framework are presented, and finally, the conclusions are drawn in **Section 4**.

## 2. Proposed Framework

**Figure 1:**
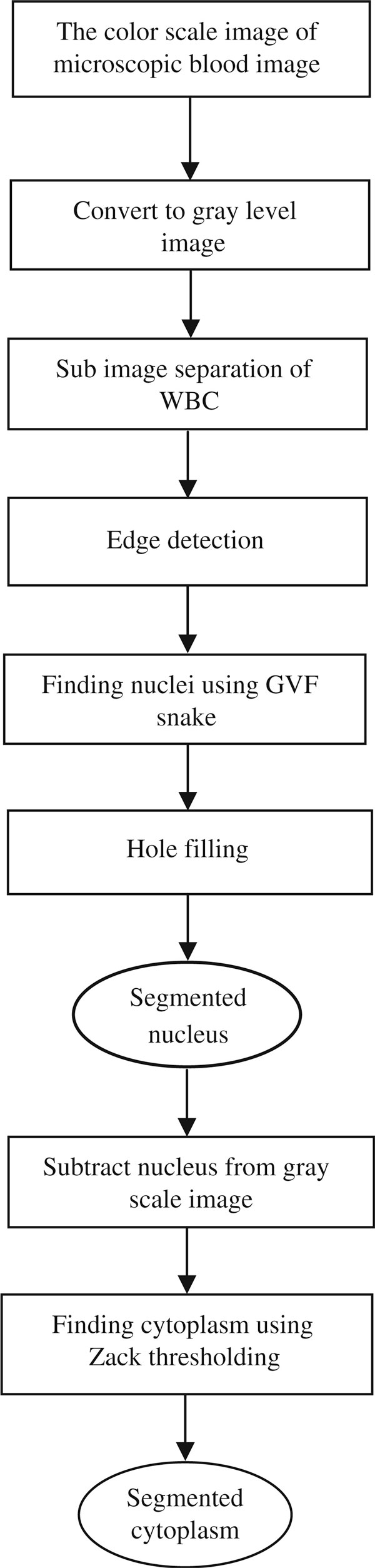
**The proposed framework of the WBC segmentation scheme**.

The goal of WBC segmentation is to separate leucocytes from other different components in blood image. A typical peripheral blood smear image consists of four components, which are the background, red cells (un-nucleated cells), white cell's nucleus, and cytoplasm. WBC appears rather darker than the background, and red cells appear in an intermediate intensity level [6]. Also, there is shape variation in cells and their nucleus. Figure 1 shows the proposed framework of the segmentation scheme. Basically, it is a two-part process of WBC segmentation into nucleus and cytoplasm after converting RGB original images to gray scale. All modules in this framework work on gray level images.

### 2.1. Nucleus Segmentation

Nuclei have variable shapes in different kinds of leukocytes. Finding a significant method for shape modeling and segmenting the nucleus has always been a challenge for scientists. Among segmentation methods, active contour models (snakes) have gained a lot of attention recently [9]. Snakes are deformable curves that can move and change their shapes to deform to boundaries of objects in an image. Curves are defined within an image domain and can move under the influence of internal forces within the curve itself and external forces derived from the image data. The internal and external forces are defined in a way that the snake conforms to an object boundary or other desired features within an image [9,10].

Two general types of active contour models have been introduced: parametric [9] and geometric active contours [11]. Geometric active contour models or geodesic snakes have been proposed to address the fact that parametric active contour models cannot resolve topological changes. For our processing scheme, the segmentation is done on sub-images, so there are no topological changes since only one object of interest exists in each sub-image. In parametric snake model [11], a traditional snake is a curve *x*(*s*) defined in **Eq.** 1 that moves through the spatial domain of an image to minimize the energy function defined in **Eq.** 2.

(1) x(s)=[x(s),y(s)],s∈[0,1]

(2) $$E=\int_{0}^{1}\left[\frac{1}{2}\left(\alpha {\left|x\prime (s)\right|}^{2}\right)+\beta {\left|x\prime \prime (s)\right|}^{2}+E_{ext}\left(x(s)\right)\right]\;ds$$

Where *α* and *β* are weighting parameters that control the snake's tension and rigidity, respectively. *x'*(*s*) and *x*"(*s*) denote the first and second derivatives of *x*(*s*) with respect to *s*. The external energy function *E*_ext_ is derived from the image so that it takes on its smaller values at features of interest, such as boundaries.

Image gradients can be used as the external and internal forces in parametric active contour models. Gradient vector flow (GVF; [11]) is a better gradient-based model due to its insensitivity to initial positions and larger capture region. GVF points toward the object boundary when addressed closed to the boundary, but varies smoothly over homogeneous image regions, extending to the image border. GVF field is defined to be the vector field *v*(*x*,*y*) (**Eq.** 3) that minimizes the energy function defined in **Eq.** 4.

(3) v(x,y)=(u(x,y),v(x,y))

(4) $$\varepsilon =\iint \left\lfloor \mu \left(u_{x}^{2}+u_{y}^{2}+v_{x}^{2}+v_{y}^{2}\right)+{\left|\nabla f\right|}^{2}{\left|v-\nabla f\right|}^{2}\right\rfloor dxdy.$$

Where *μ* is the regularization parameter, and *f*(*x*,*y*) represents edge map proceed from image *I*(*x*,*y*) as defined in **Eq.** 5. The field ∇*f* has vectors pointing toward the edges, and generally, it has a narrow capture range.

(5) $$\begin{array}{cc} f(x,y)=-E_{ext}^{i}(x,y) & i=1,2,3\;or\;4 \end{array}$$

That *E*_ext_ is an external energy designed to lead an active contour toward step edges. For the complimentary description on the variables, the reader is referred to [11]. Object boundaries play an important role in calculation of GVF. Xu and Prince defined an edge map that is larger near the edges derived from the image [11]. In this study, blood cell boundaries have been extracted using Canny edge detection [12]. By using this method, edges occurring in image would not be missed, and there would be no responses to non-edges. The step-by-step procedure of method is described as follows [13]:

1. Smoothing the image with Gaussian filter to reduce noise and unwanted details with standard deviation, *σ*.

(6) $$g(x,y)=G_{\sigma }(x,y)\times f(x,y)$$

Where

(7) $$G_{\sigma }=\frac{1}{\sqrt{2\pi \sigma^{2}}}\exp\left[-\frac{x^{2}+y^{2}}{2\sigma^{2}}\right]$$

2. Gradient calculation of *g*(*x*,*y*) using any of the gradient operators (Roberts [14], Sobel [15], Prewitt [16], etc.) to get:

(8) $$M(x,y)=\sqrt{g_{x}^{2}(x,y)+g_{y}^{2}(x,y)}$$

And

(9) $$\theta (x,y)=\tan^{-1}\left[g_{y}(x,y)/g_{x}(x,y)\right]$$

3. Threshold *M*:

(10) $$M_{T}(x,y)=\{\begin{array}{cc} M(x,y) & ifM\left(x,y\right)\rangle T\\ 0 & Otherwise \end{array}$$

Where *T* is chosen in a way that all edge elements be kept while most of the noise is suppressed. Equation 10 checks whether each non-zero *M*_T_(*x*,*y*) is greater than its two neighbors along the gradient direction *θ*(*x*,*y*). If it is, *M*_T_(*x*,*y*) will be kept unchanged; otherwise, it will be set to zero. This process is known as no maximal suppression. Next, these processes are implemented:

1. Ridge pixels are thresholded using two thresholds *T*_1_ and *T*_2_ with *T*_1_⟨*T*_2_. Ridge pixels with values between *T*_1_ and *T*_2_ are weak edge pixels, and those with values larger than *T*_2_ are strong edge pixels.

2. Edges segments in *T*_2_ are linked to form continuous edges. To do so, each segment in *T*_2_ is traced to find its end, and its neighbors in *T*_1_ are searched to find any edge segment in *T*_1_, which can bridge the gap until reaching another edge segment in *T*_2_.

By this edge detection method, central connected object boundaries that represent the nucleus are clearly obtained. In next step, GVF of the images were calculated based on **Eq.** 4 and used as internal and external forces to guide snakes to deform to nucleus boundary edges. Nucleus is the connected boundary in image and has been filled up [13] by following instruction to have a clear segmented nucleus:

Assume *f* as the marker and *g* as the mask. Marker must be a subset of mask,

(11) f⊆g

We choose the marker image *f*_m_ as below:

(12) $$f_{m}(x,y)=\{\begin{array}{ll} 1-f(x,y) & if\;(x,y)\;is\;on\;the\;border\;of\;f\\ 0 & otherwise \end{array}$$

Then, we define mask *g* in a way to represent hole filling in *f*:

(13) $$g={\left\lfloor R_{f^{C}}\left(f_{m}\right)\right\rfloor }^{c}$$

Where *R* is a reconstruction of *f*^c^ from *f*_m._

### 2.2. Cytoplasm Segmentation

By subtracting the segmented nucleus from the original sub-image, we will obtain the cytoplasm, RBC, and the background. Most of the time, RBCs appear in the image border. Looking at the gray level intensities, the cytoplasm and two other components are having almost uniform areas. Therefore, it justifies the need for segmenting these uniform components using thresholding techniques.

**Figure 2:**
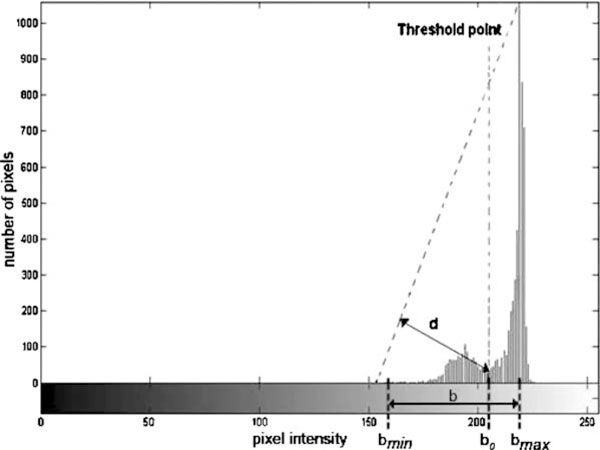
**Threshold value calculated by Zack's algorithm**.

There are many thresholding techniques available in literature [13]. Here, we set the threshold value based on Zack algorithm [17]. According to Zack's algorithm, in gray intensity histogram (*h*[*x*]) of the remaining sub-image components, a line is constructed between the highest histogram value (*h*[*b*_max_]) and the lowest histogram value (*h*[*b*_min_]), where *b*_max_ and *b*_min_ indicate the gray level values in which the histogram *h*[*x*] reaches its maximum and minimum, respectively. The distance *d* between the line and the histogram values *h*[*b*] (where *b* is the gray level values between *b*_min_ and *b*_max_) is computed for all values of *b*. The intensity value *b*_o_ where the distance *d* reaches its maximum defines the threshold value. This concept is better shown in Figure 2. Note that the histogram values representing the subtracted area have been ignored. This technique is particularly effective when the object pixels produce a weak peak in the histogram. The output from segmentation methods are shown in the following section.

## 3. Results and Discussion

This section is to assess the performance of the proposed WBC segmentation scheme. In our experiment, 20 blood images from acute leukemia cases type L2 were captured using Microscope Olympus BX51. In more details about this digital microscopy acquisition, whole system is soft imaging system with AnalySIS software. Its camera is CC-12, and magnification used ×400 for pictures. Amount of fields per slide acquired is quiet random, which is 2–3 field/slide and total slides are 10.

**Figure 3:**
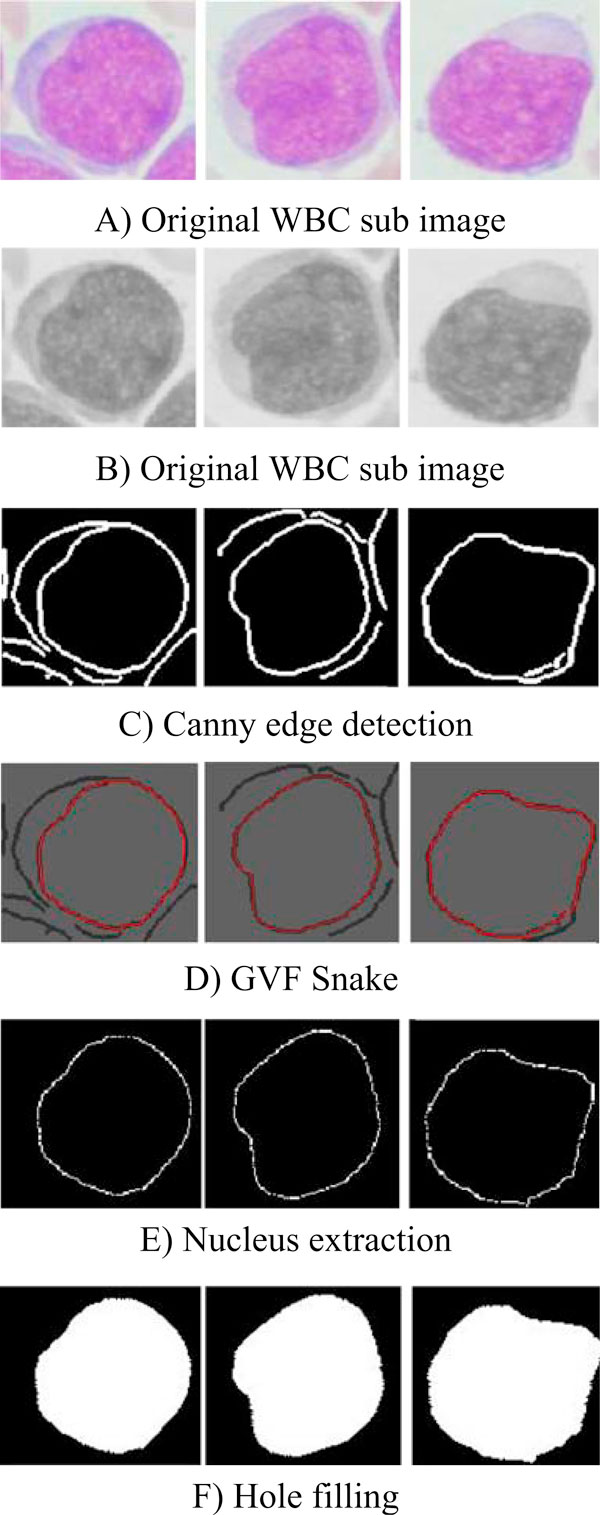
**Nucleus segmentation procedure**. **a** Original WBC sub image. **b** Original WBC sub image. **c** Canny edge detection. **d** GVF snake. **e** Nucleus extraction. **f** Hole filling.

The nucleus segmentation sequence has been shown in Figure 3a to 3f. Figure 3a shows sub-image examples of white blood cells. Figure 3b shows the RGB to gray scale converted images. Figure 3c indicates the detected edges of the same images shown in Figure 3b. Sharp changes in image brightness are very important in boundary detection. Points in the image where brightness changes significantly are often referred to as edges or edge points. As shown in Figure 3b, there is a big brightness change between nucleus and other sub-image parts (including cytoplasm, RBC, and background). So, edge detection will result in clearly separation of nucleus boundary.

GVF deformable contour was done with suitable iterations, and the final results are shown in Figure 3d. Snake algorithm finds the connected boundary that is detected in Figure 3c and it selects the nucleus. The result has been shown in Figure 3e. The connected boundaries have been filled up and shown in Figure 3f representing the nucleus of the WBC.

**Figure 4:**
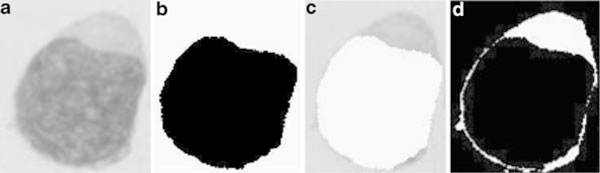
**a Original image, b segmented nucleus, c image resulting from subtracting a with b, d segmented cytoplasm**.

Figure 4 shows cytoplasm extraction result. Figure 4c represents the remaining components of the sub-image after nucleus subtraction. For getting nucleus subtraction, we refer to Figure 3f and then subtract it from WBC gray scale image shown in Figure 4a. The cytoplasm segmentation result based on the Zack algorithm is shown in Figure 4d.

Results present the advantages of our method instead of others. In nucleus segmentation, we used snake algorithm that is not related to size and color of nucleoli because there are various shapes of nucleus in a different kind of white blood cells. So, it gives high accuracy result in segmenting nucleus in any type of WBCs and in any capture illumination that cause different color space in images. Also, in a cytoplasm segmentation method in which we used the thresholding technique, background is completely segmented from other components. And, based on the difference between RBC color in blood image and cytoplasm area, thresholding value is easily segmented in the cytoplasm part. But, we prefer to use a sub-image that contains individual WBC to get a better result. This method is very simple with high speed and trustable accuracy.

By the results we got from this framework, hematologists can decide on the types of WBC and its maturation and also potentially can calculate the amount of cells in specific blood smear and finally in whole body blood. For some of the diseases like leukemia, knowledge about amount of WBCs and also their maturation is very important. In hematology science, information about size and volume of nucleus and cytoplasm is profitable. Our method gives useful information about WBC maturation status by finding the dimension of WBC components, nucleus and cytoplasm.

The framework has been done on sub-images to have easier implementation; this calls the major limitation in our method. In blood image, there are similar color scales in WBCS with some other blood particles that cause a big error in thresholding method for cytoplasm segmentation, so we individuate the WBCs in sub-image to reduce the errors. In the future, we will try to segment sub-images automatically to have a WBC segmentation process that is fully automated. The method has been applied to 20 images. We can calculate the percentage of the accuracy by evaluating WBCs' component segmentation base on the comparison of our own method and manual segmentation. We get an average accuracy of 92% for nucleus segmentation and 70% for cytoplasm segmentation. Since the cytoplasm segmentation process depends on the result of the nucleus segmentation, hence, the 8% (100 - 92%) error yielded by the nucleus segmentation affects directly on the cytoplasm extraction accuracy. Leaving this fact behind, the accuracy of the cytoplasm alone is 78%.

On average, there are about 55 WBCs in a typical blood smear image as explained before for our acute leukemia, type L2, sample blood images. Based on the results (92% accuracy for nucleus and 78% for cytoplasm), after applying the method on a sample image, there is a chance that some parts of the cytoplasm and the nucleus be missed in each WBC. This comprises the 8% and 22% errors and may show its effect on estimating the ratio of nucleus and cytoplasm only. The results show significant accuracy to be used for further analysis of blood images on detection of acute lymphoblastic leukemia or any other diseases related to WBCs.

## 4. Conclusions

This paper has demonstrated a proposed framework for segmenting white blood cells using integration of concepts in digital image processing. The proposed scheme has two parts: The nucleus segmentation part is based on morphological analysis, and the cytoplasm segmentation is based on pixel-intensity thresholding. The results show that the proposed method is able to yield 92% accuracy for nucleus segmentation and 78% for cytoplasm segmentation.
